# Tale of Three Dithienylethenes: Following the Photocycloreversion with Ultrafast Spectroscopy and Quantum Dynamics Simulations

**DOI:** 10.1021/acs.jpcb.4c04135

**Published:** 2025-01-27

**Authors:** Arkadiusz Jarota, Ewa Pastorczak

**Affiliations:** † Institute of Applied Radiation Chemistry, Lodz University of Technology, Wróblewskiego 15, 93-590 Łódź, Poland; ‡ Institute of Physics, Lodz University of Technology, ul. Wólczańska 217/221, 93-590 Łódź, Poland

## Abstract

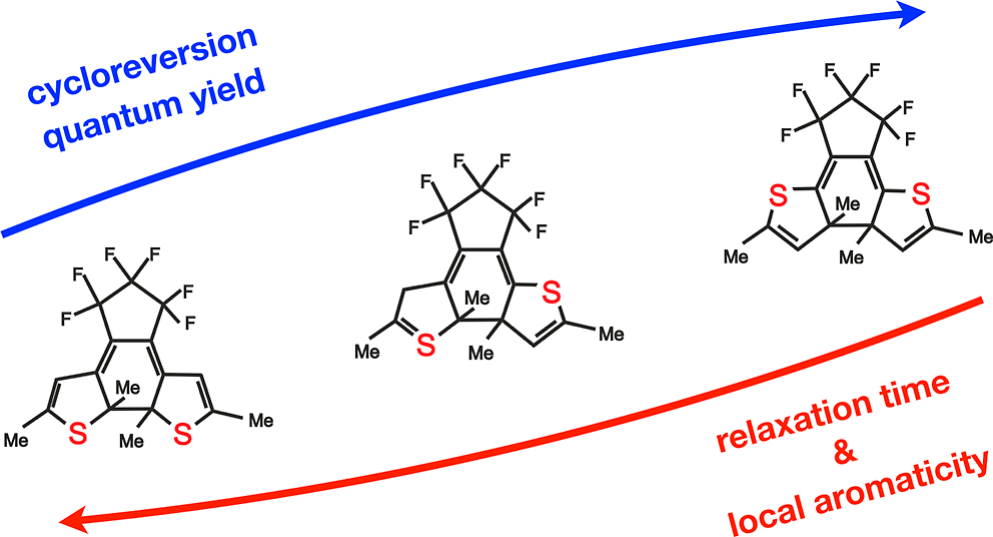

Photocycloreversion reactions of three diarylethene derivatives whose structures differ only in the placement of two sulfur atoms in the cyclopentene rings are investigated. Despite the minuscule differences between the molecules, both the yields and times of the photoreactions vary considerably. Using UV–vis and infrared femtosecond spectroscopy and quantum chemical dynamics simulations, we elucidate the relationships among the quantum yield, electronic and vibrational relaxation time, and structural properties of the dithienylethene photoswitches. We show that the local aromaticity of the molecule’s central ring could be one of the predictors of the quantum yield and the rate of cycloreversion. While from the perspective of electronic dynamics, the cycloreversion is completed within a few picoseconds at most, all three derivatives exhibit much longer (10–25 ps) nuclear rearrangement times that determine the actual times of stable photoproduct formation.

## Introduction

Photochromic switches undergo reversible structural changes upon light absorption, resulting in interchangeable chemical forms that differ by electronic absorption spectra and physicochemical parameters, including redox potentials and electrical and luminescence properties.^1^ The ability to control these properties in a repeatable manner reversibly has opened pathways for numerous potential applications.^2−8^

Recently, the family of fluorinated diarylethene (DAE) photoswitches has gathered attention due to their excellent thermal stability and high fatigue resistance, allowing for many photocycles to be performed without apparent sample decomposition.^9,10^

**Figure 1:**
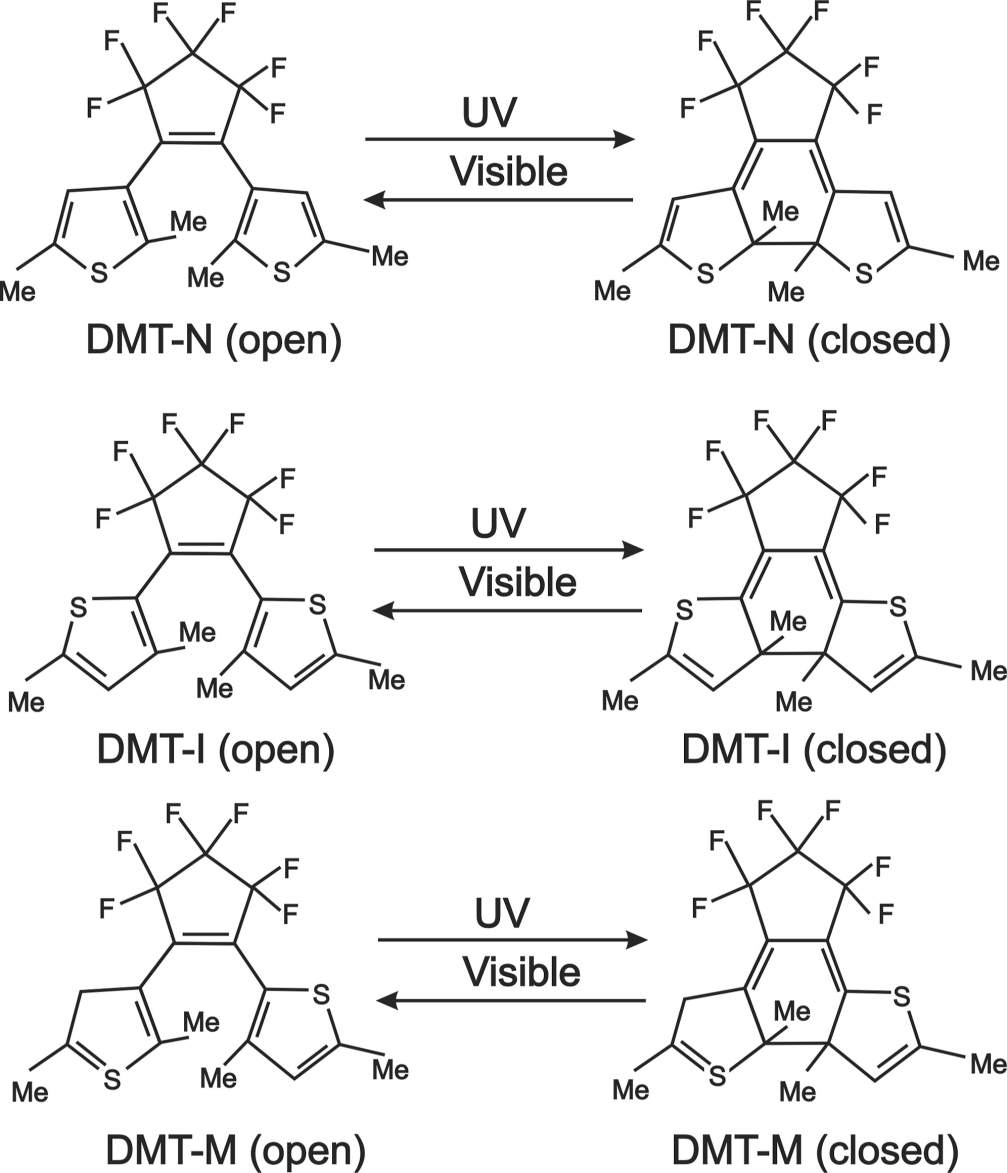
Photochromic reactions of DMT.

From an application perspective, it is important to study the relationships between the chemical structure and the quantum yield of photochromic reactions. In the case of DAE derivatives, even minuscule differences in the structure can result in significant changes in the quantum yield of the photoreactions. For example, the three molecules shown in Figure 1 feature distinct quantum yields of ring opening and closure reactions, despite the fact that they only differ in the positions of sulfur atoms in the cyclopentene rings.^11^ Specifically, when both sulfur atoms are located on the side of the molecule opposite to the fluoropentene ring, the DAE derivative exhibits a higher quantum yield of ring opening than ring closure and is called the normal (N) type (cf. top panel in Figure 1—DMT-N). If sulfur atoms occupy the side of the DAE molecule close to the perfluoropentene ring, cycloreversion occurs more efficiently than the ring closure [inverse (I)-type DAE—middle panel of Figure 1]. Finally, the quantum yields of both photoreactions may be comparable when sulfurs occupy opposite sides of the molecule [mixed (M)-type derivative, bottom panel of Figure 1].

These differences in quantum yields can be elucidated by considering the potential energy profiles of the electronic states participating in the process of electronic energy relaxation. It has been proposed that the I-type DAEs lack an energetic barrier in the *S*_1_ state that hinders cycloreversion,^12^ in contrast to N-type DAEs. Our nonadiabatic dynamics simulations^13^ confirmed that the excitation of the closed ring isomer (CRI) of DMT-I with light at wavelengths characteristic for its optical absorption (maximum around 430 nm) leads to the *S*_1_ state, from which the molecule dissipates electronic energy through a single relaxation channel, leading to a conical intersection (CI). In this CI, the DMT-I molecule has an open-ring-like geometry that favors relaxation to the open-ring form in the electronic ground state. The remarkably short electronic lifetime of *S*_1_ state (∼430 fs), determined by means of transient absorption (TA) measurements, was consistent with the computational results and supports the hypothesis of a single, efficient relaxation channel toward the ring-open form.

In this article, we further explore the correlations between chemical structure, relaxation pathways, and quantum yield of photochromic reactions. To accomplish this goal, we employ femtosecond TA probing in the UV–vis and IR spectral regions as well as quantum chemical dynamics simulations. Our focus of this work is on DMT-N and DMT-M, and we compare our results with previously published studies on DMT-I.

The rates of photochromic reactions constitute a key parameter in quantifying and assessing their usefulness in various applications. They can be determined by means of TA spectroscopy in the visible range by monitoring the change of absorbance at wavelengths associated with the substrate or photoproduct. However, the measured TA signal Δ*A* in the visible range often does not exclusively represent the concentration change of molecules involved in the photochromic reaction. The reason for this is that other physicochemical processes, such as vibrational cooling or intersystem crossing, can also contribute to the Δ*A* signal. Moreover, the ring opening/closure reactions in most cases cannot be directly followed at wavelengths in the UV range since both isomers absorb in this range. Additionally, when the electronic relaxation is completed, the photoproduct is still not in the equilibrium state and undergoes subtle structural rearrangements due to vibrational cooling. This indicates that photoreaction is completed from the point of view of the electronic structure, but it still remains unfinished considering the stability of the geometry of the molecule. While ultrafast spectroscopy in UV–vis can monitor electronic relaxation, it often struggles to definitively capture structural rearrangements. To address this limitation of UV–vis spectroscopy, we utilized probing in the IR region following UV–vis excitation. In this manner, employing time-resolved vibrational spectroscopy allows us to directly monitor the structural changes occurring during the photochromic transformations.^14−17^

## Experimental Setup

We have conducted TA experiments using a femtosecond laser setup. The setup includes a Ti/sapphire oscillator (Tsunami, Spectra-Physics, 82 MHz, 800 nm, pulse duration <100 fs) that is pumped by a diode laser (Millennia Pro, Spectra-Physics, 532 nm, 5 W). The laser pulses from the oscillator are amplified in a regenerative amplifier (Spitfire ACE, Spectra-Physics, 1 kHz, output power: 4 W), and then seed two optical parametric amplifiers (OPA, Topas Prime, Light Conversion). The pulse duration at the sample position was confirmed to be 150 fs through cross-correlation between the pump and probe pulses. The energies of the pump and probe pulses in TA experiments were adjusted to be 200 and 20 nJ, respectively.

For detection, we used a monochromator (iHR320) equipped with two photodetectors: a photomultiplier (PMTSS, Thorlabs) for visible detection and a 2 × 64 array of MCT detectors (FPAS0144, Infrared Associates) for IR detection. The signal from both visible and IR detectors was integrated using a multichannel laser pulse integrator system (FPAS0144, Infrared Associates). To detect time-resolved spectra in the UV–vis region, a white light continuum was generated by focusing the 1300 nm output from the OPA on a 5 mm thick sapphire plate and used as a probe beam. In the case of detection in the UV–vis region, time-resolved spectra were recorded by scanning a probe light using the monochromator and detection of single wavelengths one by one using PMT. For TA measurements, hexane solutions of DMT-I, DMT-M, and DMT-N in the photostationary state (PSS) were prepared by irradiation of solutions of open ring isomer (ORI) using the output from the OPA (300 nm, 15 μJ). Since both the ORI and the forming closed isomer (CRI) absorb at 300 nm, an illumination at this wavelength initiates both the ring opening and ring closure reactions. Therefore, converting all of the ORI to CRI in this way is not possible. At some point, the rates of ring opening and ring closure become equal, and the PSS is reached. All of the measurements were performed for PSSs to maximize the concentration of CRI in solution. The irradiations were performed until no spectral changes were detectable. The TA measurements in UV–vis have been performed for solutions in hexane. The time-resolved measurements with the IR probe have been performed in CDCl_3_ as hexane features numerous strong bands in studied IR region that would obscure the Δ*A* signals from DAE molecules. During the TA measurements, the sample was circulated in a flow cell (Harrick, DLC-M25) with a 630 μm spacer by using a peristaltic pump (Gilson, Minipulse 3). The polarizations between the pump and probe pulse were set at a magic angle (∼54.6°) to avoid contribution from molecular reorientations on the TA signal. The global analysis (shown in Supporting Information) has been performed using Glotaran software.^18^ The TRIR spectra were smoothed using a running average method with KOALA software.^19^

## Computational Details

To be able to compare the previously published results for the DMT-I molecule,^13^ we decided to use the same method of simulation of nonadiabatic dynamics of the *S*_1_ state for molecules DMT-N and DMT-M. Unfortunately, the calculated value of *D*_1_(MP2)^20^ diagnostic being above 0.04 for the normal derivative indicates that the single-reference methods do not adequately describe this molecule. A correct simulation of the dynamics of this molecule would have to involve a multireference method such as CASPT2 used for a quadricyclane photoswitch in Borne et al.^21^ or the semiempirical ODM2/MRCI approach frequently used for smaller DAEs by Jankowska and co-workers,^22−24^ which are currently beyond our computational capabilities.

The quantum dynamics simulation for the DMT-M was performed using the Gaussian09^25^ interfaced with Newton-X^26^ software. First, the structure of the CRI was optimized, and then normal modes were generated. We used a PBE0-D3/def2-SVP^27,28^ approach, which we have previously validated for simulating IR spectra of DAEs.^29^

Using the uncorrelated quantum harmonic oscillator distribution model, 49 trajectories were initiated in the *S*_1_ state, and then a TD-DFT in Tamm-Dancoff approximation PBE0-D3/def2-SVP dynamics study involving the four lowest electronic states (*S*_0_, *S*_1_, *S*_2_, and *S*_3_) was performed using a 0.5 fs time step. To account for nonadiabatic effects, the surface hopping method, employing Tully’s fewest switches algorithm,^30^ was used. The nonadiabatic couplings were computed only between states *S*_1_, *S*_2_, and *S*_3_ due to the unreliability of TD-DFT couplings for the ground state. We analyzed the trajectories using VMD software,^31^ up to the *S*_1_ – *S*_0_ energy gap value of 0.1 eV, before the CI was reached as recommended by Barbatti.^26,32^ The assignments of vibrational modes were performed based on comparisons between experimental spectra with theoretical spectra computed on DFT/PBE0D3 level.^29^

## Results and Discussion

**Figure 2:**
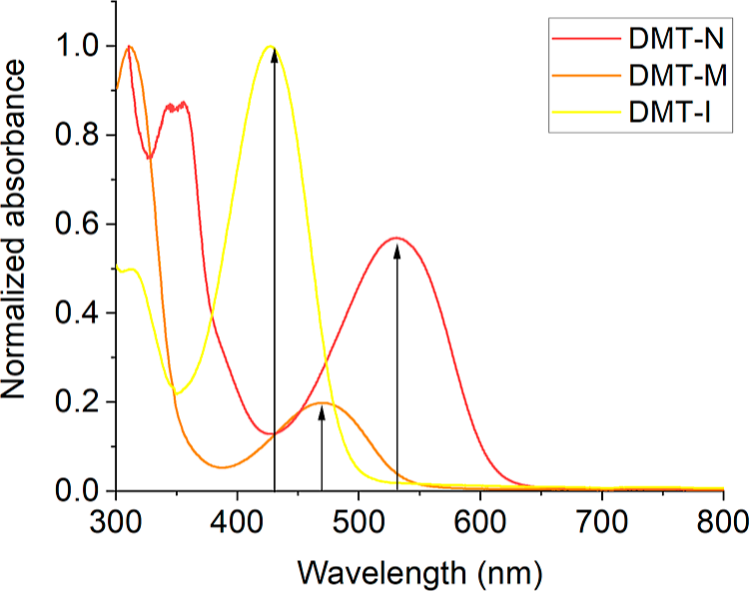
Normalized absorption spectra of studied DAEs in PSS. Arrows indicate excitation wavelengths used in time-resolved experiments.

To compare the femtosecond dynamics of DMT-I, DMT-N, and DMT-M in their PSSs, we have conducted TA measurements in which the excitation in the maximum of UV–vis absorption spectrum of the closed form (470 nm for DMT-M and 530 nm for DMT-N) was followed by a probe in UV–vis or IR probe. In the case of measurements employing UV–vis probe, only the results for DMT-M and DMT-N will be presented here since the femtosecond dynamics of DMT-I in this spectral region was already studied in our previous work.^13^ The normalized absorption spectra of the studied molecules in their PSSs with marked excitations used in pump–probe measurements are shown in Figure 2.

**Figure 3:**
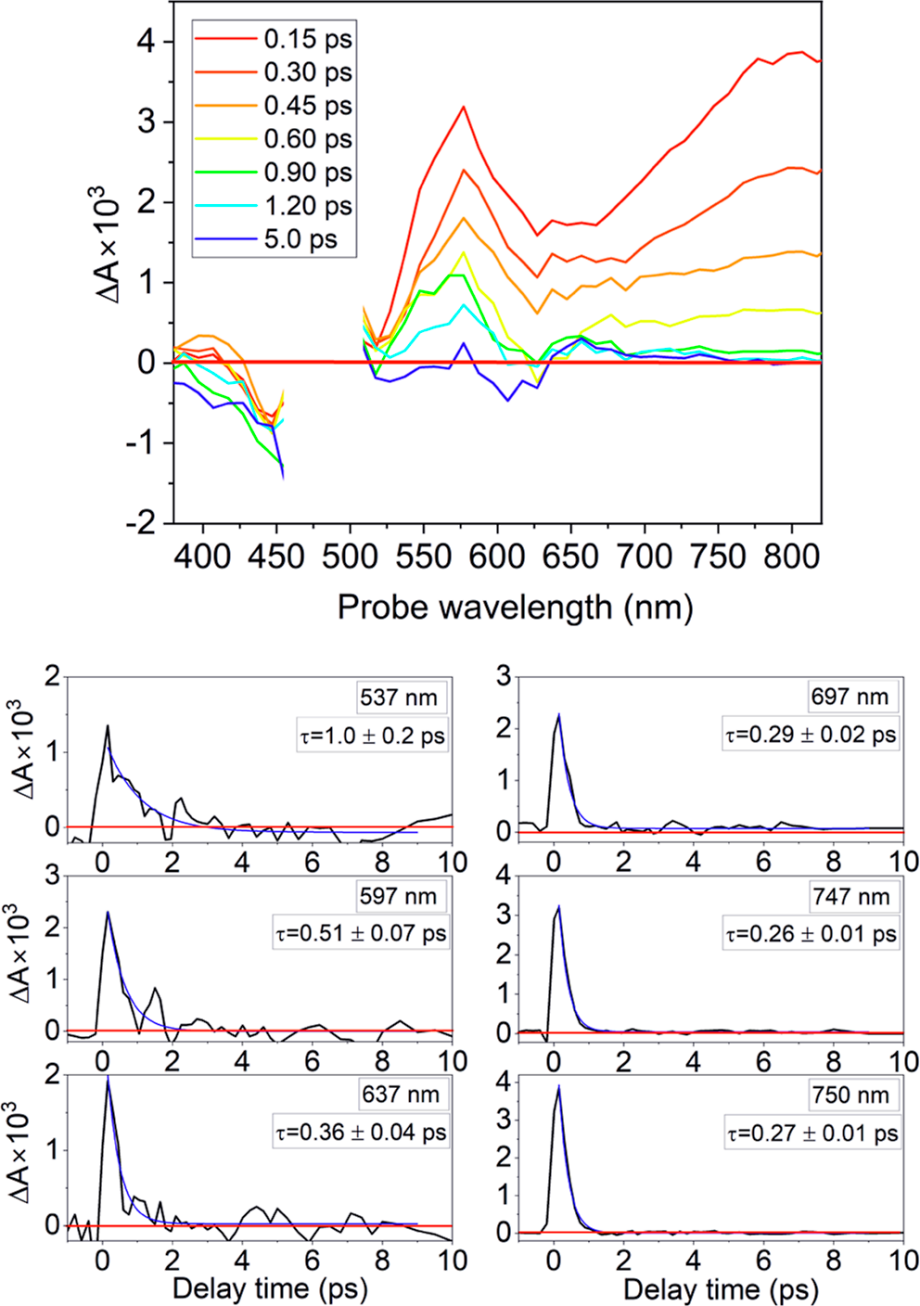
Time-resolved spectra of DMT-M in PSS, following a 470 nm excitation and selected time-traces. Straight red line represents Δ*A* = 0. Blue lines are single exponential fittings of Δ*A* signals.

The time-resolved spectra recorded for DMT-M and DMT-N using a UV–vis probe, together with selected time-traces, are presented in Figure 3. In these spectra, the Δ*A* signals around 470 nm for DMT-M and 530 nm for DMT-N are excluded from the analysis due to strong scattering light coming from the pump beam.

The time-resolved spectra of DMT-M show a broad positive band for probe wavelengths between 520 and 820 nm. As the DMT-M has the electronic ground state absorption only up to around 630 nm, the ground state has impact only on the Δ*A* signals in the high-energy edge of the 520–820 nm region. The Δ*A* signals in this spectral range should be therefore assigned to excited state absorption (ESA) occurring from the *S*_1_ electronic state to higher excited states (*S*_1_ → *S*_*n*_ absorption). By fitting the time-trajectories of Δ*A* signals with single exponential decays, we were able to determine the electronic lifetime of the *S*_1_ state. The selected time-traces and relevant fittings are presented in Figure 3, while the full set of determined time-constants for all probe wavelengths is shown in Figure S1. The determined time constants take values from 1.0 ± 0.2 ps at 537 nm to 0.36 ± 0.01 ps at 637 nm. The higher values of time constants for shorter wavelengths may be attributed to the more pronounced contribution from the vibrational relaxation in the ground state. For probe wavelengths in the range of 630–820 nm, the values of time constants change only slightly, taking values from 0.36 ± 0.01 ps at 637 nm to 0.28 ± 0.01 ps at 820 nm, with an average value of 0.30 ± 0.02 ps. We assign this value to the lifetime of the *S*_1_.

**Figure 4:**
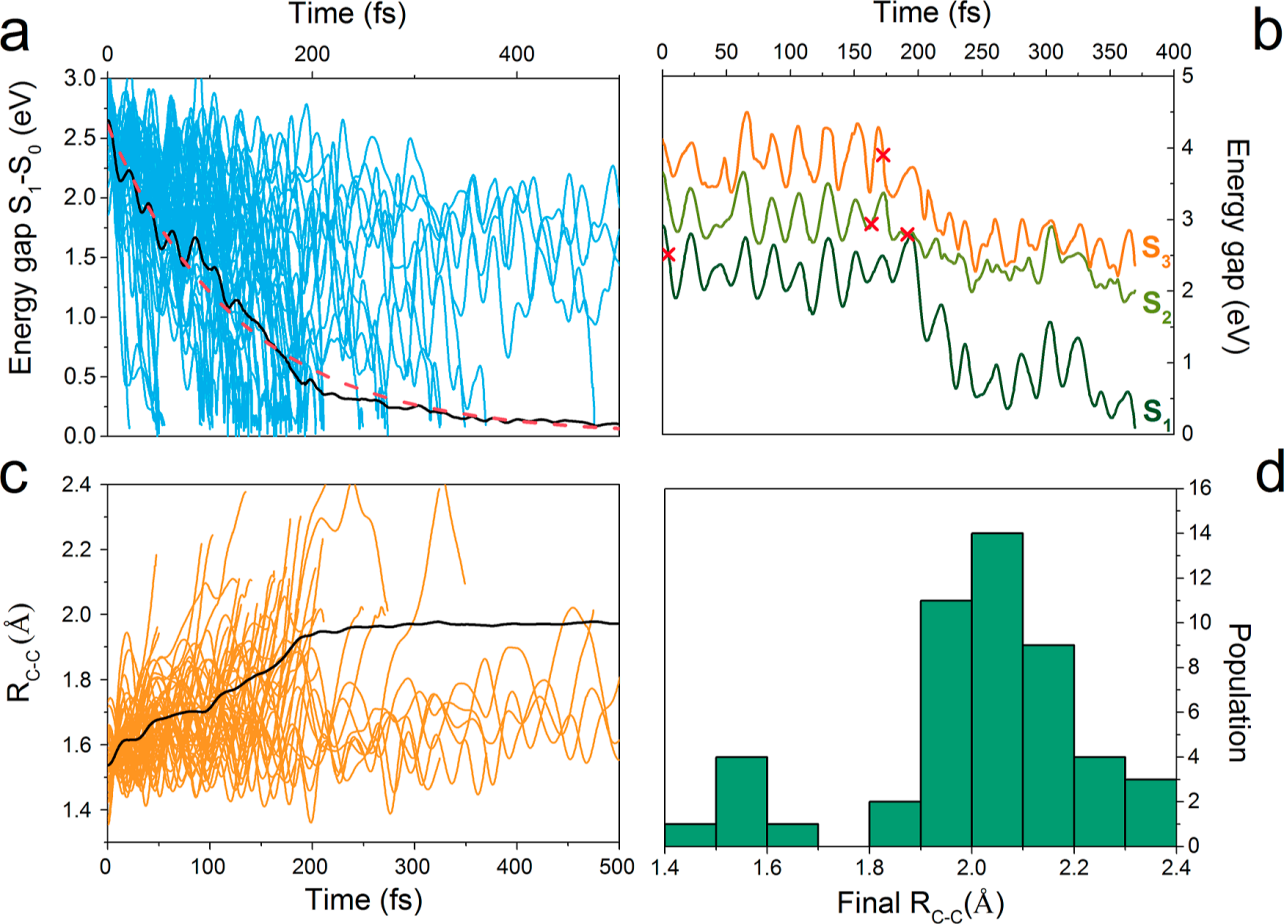
(a) Top left panel: energy gap between the ground state and the *S*_1_ state for 49 trajectories of the DMT-M molecule. Solid black line represents the average gap from 49 trajectories and dashed red line represents the exponential fit for the average gap. (b) Top right panel: *S*_1_, *S*_2_, and *S*_3_ energies (relative to the ground state energy) for an example trajectory. Red crosses represent the times of surface hopping. (c) Bottom left panel: temporal evolution of the C–C bond length involved in ring opening/closure reactions in DAE-M for 49 trajectories. Solid black line represents the mean average of C–C values from all 49 trajectories. (d) Bottom right panel: histogram of final C–C bond lengths for all trajectories.

The quantum chemical computations confirm the short relaxation time of DMT-M. The exponential decay curve fitted to the mean average energy gap (Figure 4a) predicts the time of relaxation from the *S*_1_ electronic state to the CI τ_CI_ ≈ 129.0 ± 1.6 fs.

This value is similar to the value obtained for DMT-I (τ_CI_ ≈ 120 fs). The energy gap diminishing is paralleled by the changes in the value of the C–C bond length between the central reactive carbons *R*_CC_ (cf. Figure 4b). The mean average of the final *R*_CC_ is 2.0060 ± 0.0085 Å, which is closer to the ORI form of the molecule. That being said, some of the trajectories end up in a geometry that is visibly a CRI one (Figure 4b,d). The histogram of the final *R*_CC_ has two modes: one around 2.05 Å, corresponding to an ORI, and the other, much lower, at 1.55 Å. The ratio of the number of trajectories ending up with *R*_CC_ around 2.05 Å to the number of all trajectories, 0.92, is a slightly lower number than the one obtained for the DAE-I molecule (1.00, cf., Table 1) but, since in both cases, the simulations comprised relatively small numbers of trajectories, the difference can be attributed to the uncertainty of computation. The similarity of these values of DAE-I and DAE-M is consistent with the similarity of quantum yields of cycloreversion obtained by Uchida,^11^ cf., Table 1. However, these values cannot be straightforwardly interpreted as the CI branching ratios of cycloreversion or as the approximate quantum yields of the reaction. First, the dynamics simulation is conducted until the molecule reaches the CI (notice, Table 1, that this is reflected by the difference in time constants obtained experimentally, τ_exp_, and the ones obtained computationally, τ_CI_). Even though the molecular geometry is close to the ORI form when it approaches the CI, it does not mean that it will stay open once it crosses the CI, just that the CI occurs for a geometry with a large *R*_CC_ distance. The fraction χ_CI_ = 0.92 can therefore only be interpreted as the fraction of trajectories reaching the CI of interest.

**Table 1:** Essential Parameters of the Cycloreversion Reaction in DMT-I, DMT-M, and DMT-N

	DMT-I	DMT-M	DMT-N
τ_CI_ (fs)	120^13^	129.0 ± 1.6	
τ_exp_ (fs)	590 ± 40	300 ± 20	2600 ± 300
experimental vibrational relaxation time (determined in the range 1250–1300 cm^–1^) (ps)	25.5 ± 1.3^13^	24.3 ± 1.5	10.3 ± 1.2
final *R*_CC_ (Å)	1.942 ± 0.087	2.0060 ± 0.0085	
χ_CI_	1.00	0.92	
φ_o_	0.58^11^	0.57^11^	0.13^11^
Shannon aromaticity index (×10^7^) of the central ring	9.6	5.8	2.0

When trying to predict the quantum yield of the cycloreversion, we have to also keep in mind that a dynamics simulation reflects an ultrafast process triggered by short-impulse excitation of the DAE molecule. On the other hand, the result of a quantum yield measurement using a stationary source of light must be an average of a few to a few dozens of relaxation cycles. Hence, any byproduct buildup that appears in the reaction can impair the accuracy of the measurement.

The emergence of such a byproduct was not only proposed^33^ but also observed^34,35^ for DAE molecules. In line with this observation, our computations show the possibility of formation of the byproduct, since two of the trajectories finish in a geometry consistent with the breaking of a C–S bond in one of the side rings, which is the first step toward the byproduct formation.

The number of surface hopping points in the simulation of DMT-M dynamics (51), as opposed to zero such points in DMT-I, suggests a much richer structure of potential energy surfaces of states *S*_1_, *S*_2_, and *S*_3_. We show an example of a trajectory with such hopping points in Figure 4c and included all the surface hopping point geometries in the Supporting Information, since it might assist in the search for conical seams and intersections of DAE-M’s higher electronic states.

In Table 1, we also show the value of the Shannon aromaticity index^36^ of the central ring, which quantifies the local aromaticity in the ring. The lower the value of this parameter, the more aromatic the system and, consequently, the higher the electron delocalization. Therefore, as seen in Table 1, the rise of cycloreversion quantum yield correlates with the decrease of electron delocalization.

For position isomers, such as the central rings of all three studied DAE molecules, it was shown that local aromaticity correlates with the isomer stability.^37^ Faster and more efficient cycloreversion would therefore logically correspond to lower stabilities of the C–C bond in the central ring of the DAEs.

**Figure 5:**
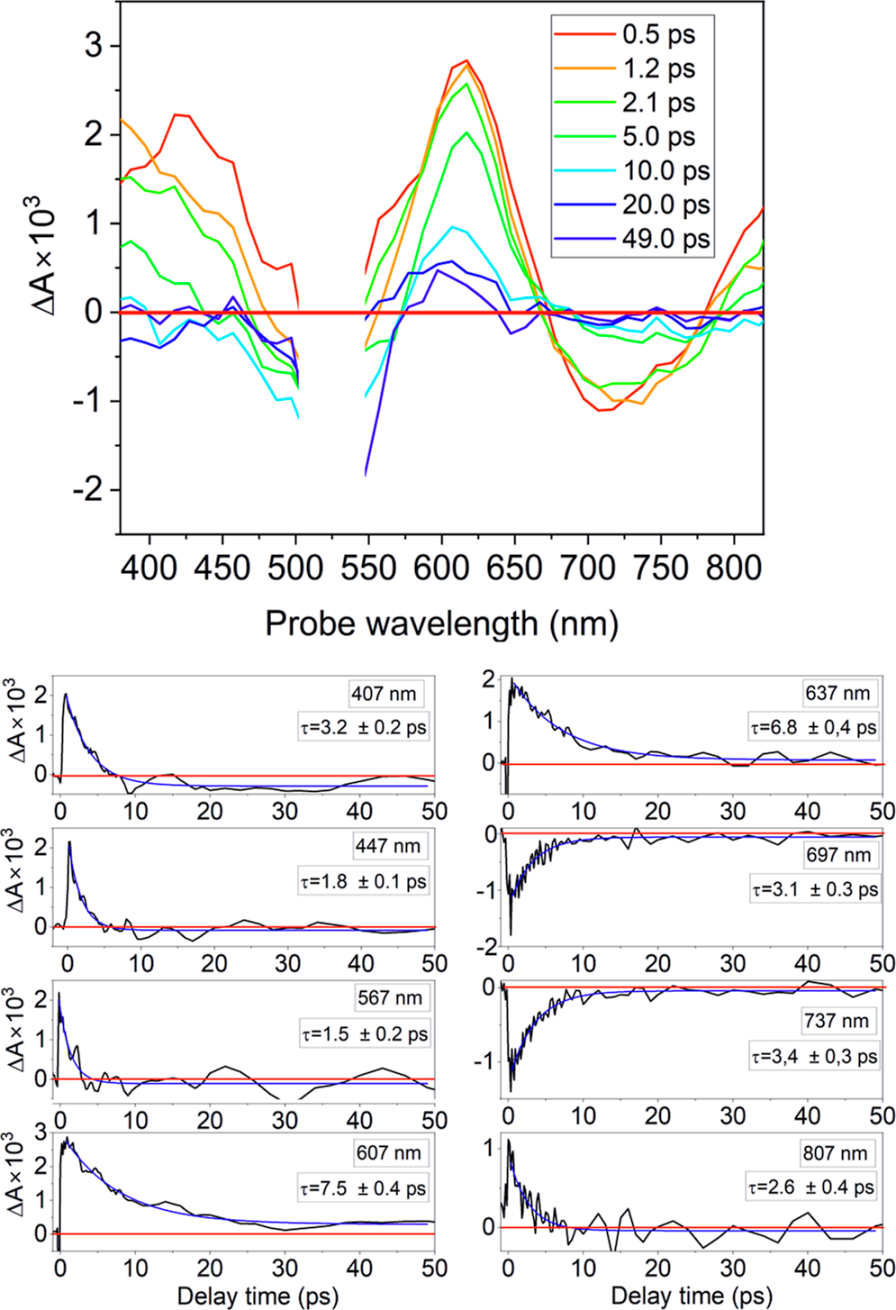
Time-resolved spectra of DMT-N in PSS following a 530 nm excitation and selected time-traces. Straight red line represents Δ*A* = 0. Blue lines are single or double exponential fittings of Δ*A* signals.

DMT-N exhibits remarkably different kinetics compared with DMT-I and DMT-M. The relevant time-resolved spectra and the corresponding time traces with their single exponential fittings are shown in Figure 5.

The positive bands observed in the time-resolved spectra in the ranges of 380–480, 580–670, and over 780 nm are dominated by ESA. Below 580 nm, DMT-N exhibits strong electronic ground state absorption, and therefore, ground state bleaching is expected to contribute to the Δ*A* signal in this spectral range. The negative Δ*A* signals that appear in the range 670–780 nm should be assigned to stimulated emission, as this is the only possible negative contribution in this region. This assignment is confirmed by the presence of a weak fluorescence band in the 670–780 nm region during stationary measurements. Another negative band appears in the range 500–560 nm due to the presence of ground-state depletion. However, the Δ*A* signals in this range are overwhelmed by the excitation pulse centered at 530 nm, which hinders a more detailed analysis.

The time constants determined in the spectral ranges 380–480 and 670–780 nm have similar average values, 2.2 ± 0.2 and 2.6 ± 0.3 ps, respectively. In the range of 570–660 nm, we observe an apparent rise of the time constant with an average value of 7.1 ± 0.6 ps. Since in this range, we can observe the *S*_0_ dynamics (the *S*_1_ state is very short-lived), and the time scale matches the typical vibrational relaxation processes, we tentatively assign this constant to the vibrational cooling of the molecule in the ground electronic state. As the time constant 2.6 ± 0.3 ps represents the range not influenced by the dynamics of the ground state, we can assign it to the lifetime of the electronic excited state *S*_1_.

While the TA signals obtained in the UV–vis region confirm the time scales of the electronic relaxation predicted by the simulations, they contain overlapping contributions from both the electronic and the vibrational relaxation, as well as from the photochromic reaction, which means they are not straightforward to interpret.

To gain a better insight into what happens after the molecules relax from their electronically excited states, we decided to follow the reactions using an IR probe covering the spectral range of 1200–1700 cm^–1^ since changes in this region are not obscured by the contributions from the electronic relaxation.

**Figure 6:**
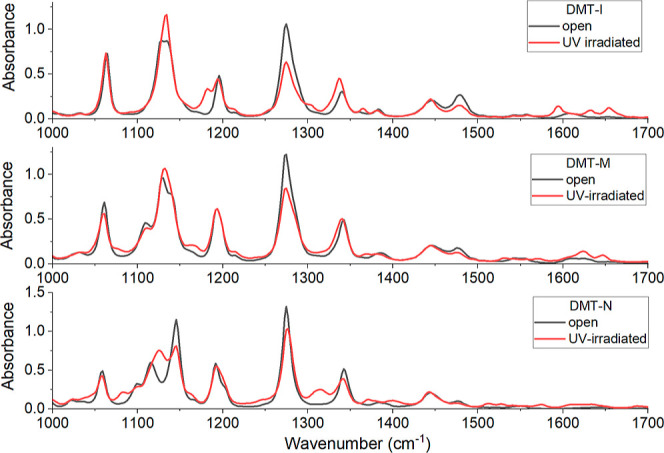
Stationary IR spectra of ORI DAE (black line) solutions and of the mixture of the ORI and CRI (red line), obtained through irradiation of the ORI solution with continuous light at 300 nm.

To identify the spectral changes occurring during a ring–opening reaction, let us first look at the stationary IR spectra of ORI DAE solutions and of the mixture of ORI and CRI, obtained through irradiation of the ORI solution with continuous light at 300 nm (see Figure 6). Even though the spectra before and after irradiation largely overlap, still, spectral changes visibly occur at certain wavenumbers. At these wavenumbers, we can track the photochromic reactions in our time-resolved experiments. The most pronounced changes for all three molecules occur in the spectral region 1250–1300 cm^–1^, where the absorbance drops significantly upon UV illumination. The vibrational modes appearing in this region majorly involve distortions of the central reactive ring and the cyclofluoropentene ring for both open and closed isomers (cf., Figures S6–S8 in Supporting Information illustrating the geometrical changes during molecular vibrations in the 1250–1300 cm^–1^ region for IR spectra computed at DFT/PBE0D3 level^29^)

**Figure 7:**
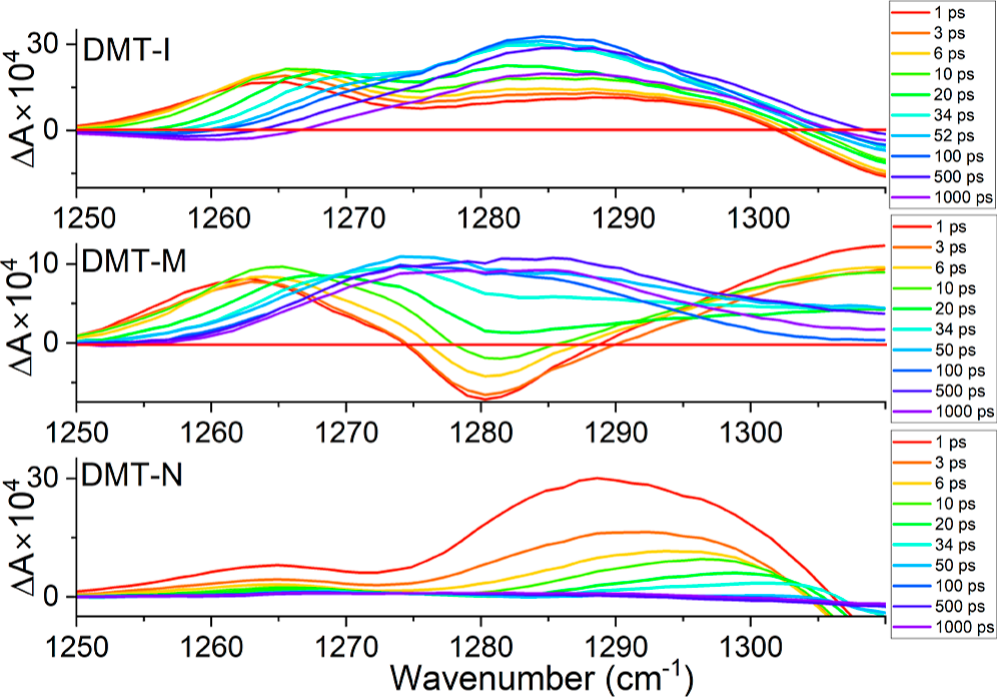
Time-resolved IR spectra of DMT-I, DMT-M, and DMT-N in PSSs following excitation at 430, 470, and 530 nm, respectively.

**Figure 8:**
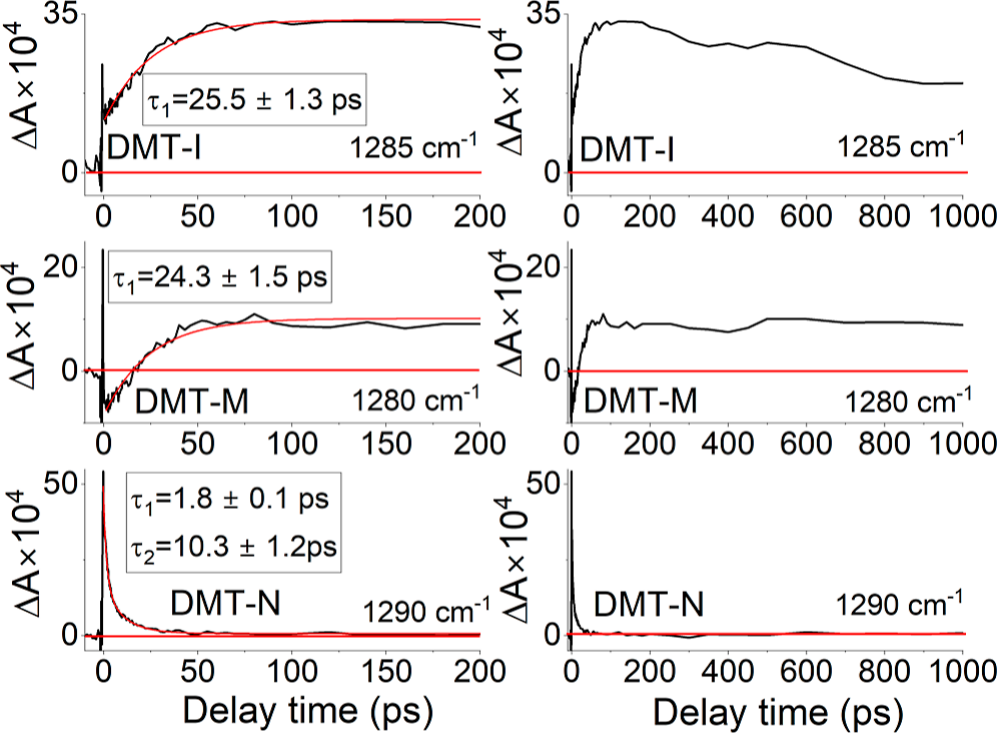
Selected time-traces from time-resolved IR spectra of DMT-I, DMT-M, and DMT-N in PSSs following excitation at 430, 470, and 530 nm, respectively.

In the case of DMT-I, the representative band with a maximum at 1285 cm^–1^ appears in the time-resolved spectra (cf. top panel of Figure 7). The intensity of this band increases with the increase of the delay time between pump and probe pulses. The corresponding kinetics at 1285 cm^–1^ (top panel of Figure 7) can be fitted with a single exponential function with a time-constant of 25.5 ± 1.3 ps (cf. top left panel of Figure 8). It is followed by a much slower decaying component whose time constant cannot be reliably determined as its duration exceeds the limitations of our delay line (i.e., 1200 ps). Importantly, for the longest recorded delay time, the Δ*A* signal remains positive, which is consistent with the increase of absorbance attributed to the ring–opening reaction initiated by UV irradiation, as observed in stationary IR spectra (cf. Figure 6). It implies that the cycloreversion contributes to the rise of the Δ*A* signal at 1285 cm^–1^ with a time constant of 25.5 ± 1.3 ps. It should be noted that the experiments were performed in a PSS containing both open and closed isomers. However, only the newly formed ORI molecules should contribute to the measured Δ*A* signal.

Photochromic reactions of DAEs proceed from the *S*_1_ excited state.^9^ Therefore, the lifetime of the *S*_1_ state can serve as a measure of the reaction’s rate. However, after returning to the ground state, geometrical changes associated with the reaction can still occur and may accompany the molecule’s thermalization. This can lead to different results for the reaction rate depending on whether we track electronic or vibrational relaxation. For DMT-I, the time constant determined from time-resolved IR experiments, 25.5 ± 1.2 ps, is much larger than the electronic lifetime of the *S*_1_ state of CRI, which was determined to be below 1 ps.^13^ This suggests that in the case of DMT-I, even though the photochromic reaction primarily occurs within the electronic lifetime of the reactant, the resulting photoproduct is initially in an unstable form, requiring subsequent geometrical rearrangements to reach equilibrium in the ground state. A similar observation was made for the DMPT-FSCP DAE using femtosecond stimulated Raman scattering spectroscopy by Pontecorvo et al.^38^ In that case, the formation of the open-ring photoproduct was reported to be completed within a few hundred femtoseconds, based on an analysis of the rise in the TA probed at 560 nm, a wavelength characteristic of the photoproduct. However, when considering the rise in the intensity of the ethylenic stretching mode at 1503 cm^–1^, characteristic of the CRI, the time constant for ring closure was determined to be 18 ps. On the other hand, e.g., Sotome et al. observed a similar time scale (approximately 13 ps) for both electronic relaxation and the formation of vibrational bands attributed to the ORI of the DAE derivative referred to as BT.^17^ This discrepancy in the literature implies a strong relationship between the molecular structure and the time scale of the electronic relaxation process.

For DMT-M, within the spectral region of 1250–1320 cm^–1^, we observed results similar to those obtained for DMT-I. The time-resolved spectra of DMT-M feature a vibrational band with a maximum at 1280 cm^–1^ (middle panel of Figure 7). Fitting the rise of this Δ*A* signal with a single-exponential time-constant Δ*A* signal results in a time constant of 24.3 ± 1.5 ps (bottom panel of Figure 8), which is very similar to that observed for DMT-I. Unlike in DMT-I, the initial sign of Δ*A* signal is negative. As the kinetics evolves, the Δ*A* signal rises and its sign becomes positive, reaching a constant value at around 80 ps. The constant positive Δ*A* value observed at longer delay times, similarly to DMT-I, indicates that a portion of the reactant is permanently converted to the ORI. Unlike for DMT-I, in the case of DMT-M, we have not observed a slower component exceeding the available delay time range.

Finally, DMT-N, similarly to DMT-I and DMT-M, has a vibrational band in the 1250–1300 cm^–1^ region with a maximum at 1290 cm^–1^ at initial delay times (bottom panel of Figure 7). However, the kinetics observed for DMT-N exhibits distinct features compared to two other derivatives. First, the time trace at 1290 cm^–1^ (bottom-left panel of Figure 8) cannot be well fitted with a single-exponential function. Instead, a two-exponential function was employed, resulting in time constants of 1.8 ± 0.1 and 10.3 ± 1.2 ps. The shorter time constant (1.8 ps) cannot be unambiguously assigned to a specific process. However, given its time scale, it likely corresponds to nonlinear effects associated with the temporal overlap of the pump and probe pulses around 0 fs. Similar signal spikes are also observed at other probe wavelengths for all three molecules. Unlike in the other two cases, for DMT-N, the initial signal spike cannot be easily excluded from the fitting by starting the fit after the spike. Additionally, while the time traces recorded for DMT-I and DMT-M exhibit a rise in the Δ*A* signal at early delay times in this spectral region, the Δ*A* signals for DMT-N show a decay throughout the entire recorded delay time range. In contrast to DMT-I and DMT-M, for DMT-N, we observe the decay of the Δ*A* signal rather than its rise, and the value of Δ*A* = 0 is reached after the relaxation is completed. Hence, in the case of DMT-N, unlike for the other two DAE derivatives, we do not observe a signature indicating the presence of molecules permanently converted to the ORI.

The primary reason for the observed dynamics of DMT-N being significantly different from those of DMT-I and DMT-M is that DMT-N is a normal-type DAE with a significantly lower quantum yield for the cycloreversion reaction compared to the other derivatives. Therefore, the contribution from the photochromic reaction is minor in the case of DMT-N, as also evidenced by the smaller changes observed in the stationary IR spectra. For DMT-N, the longer time constant should therefore be attributed exclusively to the vibrational relaxation of the CRI, without any contribution from cycloreversion.

Additionally, in the time-resolved spectra, we observe a population exchange from 1265 cm^–1^ species to 1285 cm^–1^ for DMT-I and from 1263 to 1280 cm^–1^ for DMT-M. However, this trend is not apparent in the DMT-N molecule. To disentangle the effects of various concurrent phenomena on the spectra, we have calculated the decay-associated spectra and evolution-associated spectra for all three molecules to investigate spectral changes in the 1250–1320 cm^–1^ region. The results are presented in the Supporting Information (Figures S3–S5). The time-resolved spectra show the following: for DMT-M and DMT-N, a short component, with time constants of 200 fs and 2.3 ps, correspondingly, attributed to the early dynamics involving the coherent artifact and the following solvent reorientation; for all three molecules, a middle component, with time constants between 10 and 40 ps, corresponding to the vibrational dynamics; and a long-lasting component, with a time constant greater than 1000 ps, which only appeared for the inverse and the mixed derivatives. Since the stationary ORI and CRI absorption spectra differ in this range, we, similarly to Sotome et al.,^17^ associate this longest component with the formation of a new species, i.e., the ORI. The appearance of this nanosecond-long time-constant is therefore the signature of the ring–opening reaction.

## Conclusions

Combining ultrafast TA spectroscopy and quantum chemical dynamics simulations, we have elucidated the electronic and vibrational dynamics in a set of fluorinated DAEs containing a thiophene ring, namely, DMT-I, DMT-M, and DMT-N. These three molecules have been selected based on their similar structures and markedly different quantum yields for ring opening/closure reactions. The experimentally determined *S*_1_ lifetimes for DMT-I and DMT-M (590 ± 40 and 300 ± 20 fs, respectively) accompany high quantum yields of ring opening reactions (0.58 and 0.57, respectively). On the other hand, DMT-N features a much longer *S*_1_ lifetime (2.6 ± 0.3 ps) that accompanies a much smaller quantum yield (0.12). Quantum chemical dynamics simulations have confirmed a fast relaxation of DMT-M and DMT-I through a single relaxation channel. While for DMT-I,^13^ all trajectories finished in the CI responsible for the cycloreversion reaction, for DMT-M, that fraction was slightly lower, i.e., 92%, with a few trajectories ending up in geometries consistent with the beginning of a reaction creating a known byproduct of a DAE photocycloreversion. State *S*_1_ of the DMT-N molecule turned out to have a multireference character and cannot be correctly described at the same level of theory as the corresponding states of the DMT-I and DMT-M molecules. This is an interesting problem for future research.

Time-resolved experiments performed by employing a UV–vis pump and an IR probe were able to capture the signatures of the cycloreversion. For mixed and inverse derivatives (DMT-M and DMT-I) the presence of a long-lasting Δ*A* signal in the experiments with an IR probe indicates the completion of the photoreaction. These persistent signals typically appear at delay times longer than 10–25 ps, while the electronic dynamics for DMT-I and DMT-M is completed in times below 1 ps. A distinct picture is observed for DMT-N. For this molecule, we have not observed a long-lasting Δ*A* signal in the 1250–1300 cm^–1^ spectral region, indicating that the concentration of the ORI is too low to manifest in the time-resolved experiment. This is consistent with the low cycloreversion quantum yield (0.13) of DMT-N. The interpretation of the results obtained using the IR probe is somewhat ambiguous due to the presence of two simultaneous processes: the thermalization of the system and the photochromic reaction [unlike, for example, in the work of Jean-Ruel et al., who used a more direct method to track nuclear motions associated with the photochromic reaction: UV pump and X-ray probe—femtosecond crystallography].^9,39^ Interestingly, the values of the quantum yield of the cycloreversion in the three molecules correlate with the values of the Shannon aromaticity index calculated for the central ring of each molecule. This relationship between quantum yields and aromaticity should be investigated further for a larger set of DAEs.

This work illustrates the caveat of assessing the “speed” of a photoswitch. The most intuitive measures are the electronic relaxation times of cycloreversion and cyclization.^13,21,40,41^ In practice, the key parameter is the time between initiating both the cyclization and the reverse reaction that ensures maximum yields for both reactions. The influence of vibrational cooling and geometrical rearrangements on the reaction’s yield is an individual property of the molecule. For a phenylthiophene-based derivative, DMPT, the increase of cycloreversion reaction yield correlates with the rise in temperature.^42^ Starting the cycloreversion from a vibrationally hot substrate can facilitate overcoming a barrier in the *S*_1_ state and enhance the reaction yield. Therefore, the vibrational relaxation in the ground state can impact the quantum yield of the following ring opening reaction.

While the cycloreversion electronic relaxation time is one of the key parameters for a photoswitch, as it seems to directly correlate with the reaction yield, for practical applications, the vibrational relaxation time is just as important to determine. Time-resolved UV–vis pump/IR probe spectroscopy is a convenient (albeit not sole^42^) technique for this purpose.

## Data Availability

The output files from Gaussian for the vibrational spectra calculations of CRIs of DMT-I, DMT-M, and DMT-N.

## Supporting Information Available

Vibrational spectra of CRIs of DMT-I, DMT-M, and DMT-N calculated at DFT/PBE0-D3 level (ZIP)
Probe wavelength-dependence of time constants determined in time-resolved experiments in UV–vis spectral range for DMT-M and DMT-N, results of global analysis of TA measurements of DMT-I, DMT-M, and DMT-N in the experiments employing UV–vis pump and IR probe, graphical representations of molecular vibrations based on theoretical vibrational frequencies calculations at DFT/PBE0-D3 level, and surface hopping point geometries (PDF)

## Abbreviations

DAE: diarylethene
DMT-N: normal derivative of DMT molecule
DMT-I: inverse derivative of DMT molecule
DMT-M: mixed derivative of DMT molecule
PSS: photostationary state
CI: conical intersection
TA: transient absorption
OPA: optical parametric amplifier
PMT: photomultiplier tube
TD-DFT: time-dependent density functional theory
ESA: excited state absorption
ORI: open ring isomer
CRI: closed ring isomer
SE: stimulated emission
