# Supplementary material for: Tale of Three Dithienylethenes: Following the Photocycloreversion with Ultrafast Spectroscopy and Quantum Dynamics Simulations
Source: J Phys Chem B. 2025 Jan 27;129(5):1605–13. doi: 10.1021/acs.jpcb.4c04135 (PMC11808639; doi:10.1021/acs.jpcb.4c04135)
Supplement: Supplementary file 2 — jp4c04135_si_002.pdf [file jp4c04135_si_002.pdf]

Electronic Supporting Information

for

The Tale of Three Dithienylethenes: Following the Photocycloreversion with Ultrafast Spectroscopy and Quantum Dynamics Simulations

Arkadiusz Jarota[a], Ewa Pastorczak[b]

[a]*Institute of Applied Radiation Chemistry, Lodz University of Technology, Wróblewskiego 15, 93-590 Łódź, Poland*

[b]*Institute of Physics, Lodz University of Technology, ul. Wólczańska 219 90-924, Łódź, Poland*

1. Wavelength-dependence of time constants determined in time resolved experiments in Uv-Vis spectral range for DMT-M and DMT-N.

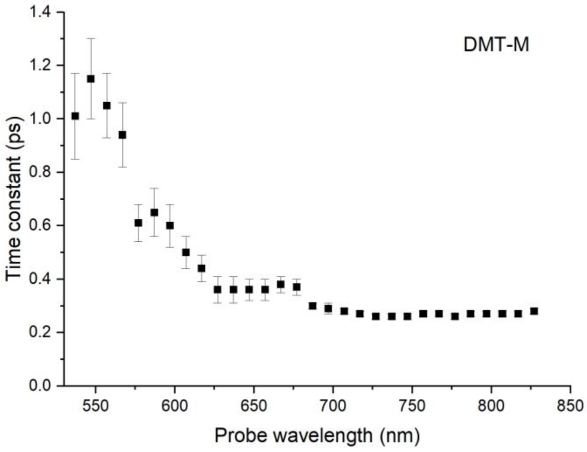

Fig. S1 Wavelength-dependence of time constants determined in time resolved experiments in UV-Vis spectral range for DMT-M.

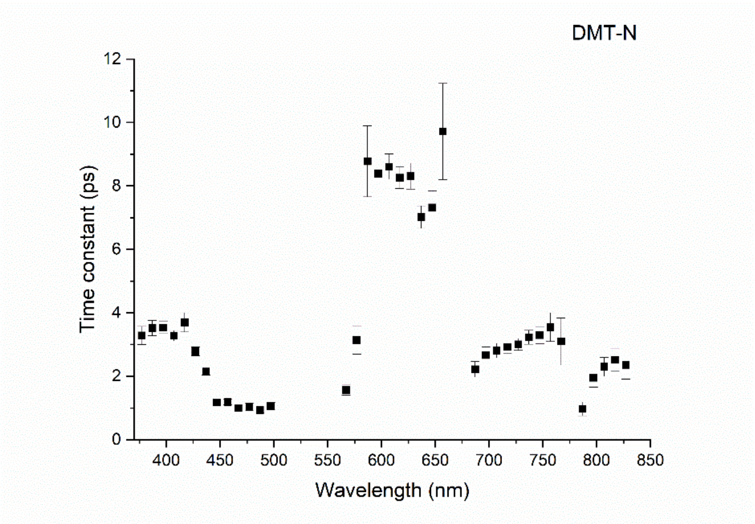

Fig. S2 Wavelength-dependence of time constants determined in time resolved experiments in Uv-Vis spectral range for DMT-N.

2. Global analysis of transient absorption measurements of DMT-I, DMT-M, and DMT-N. The analysis has been performed using Glotaran software.[1]

a) DMT-I

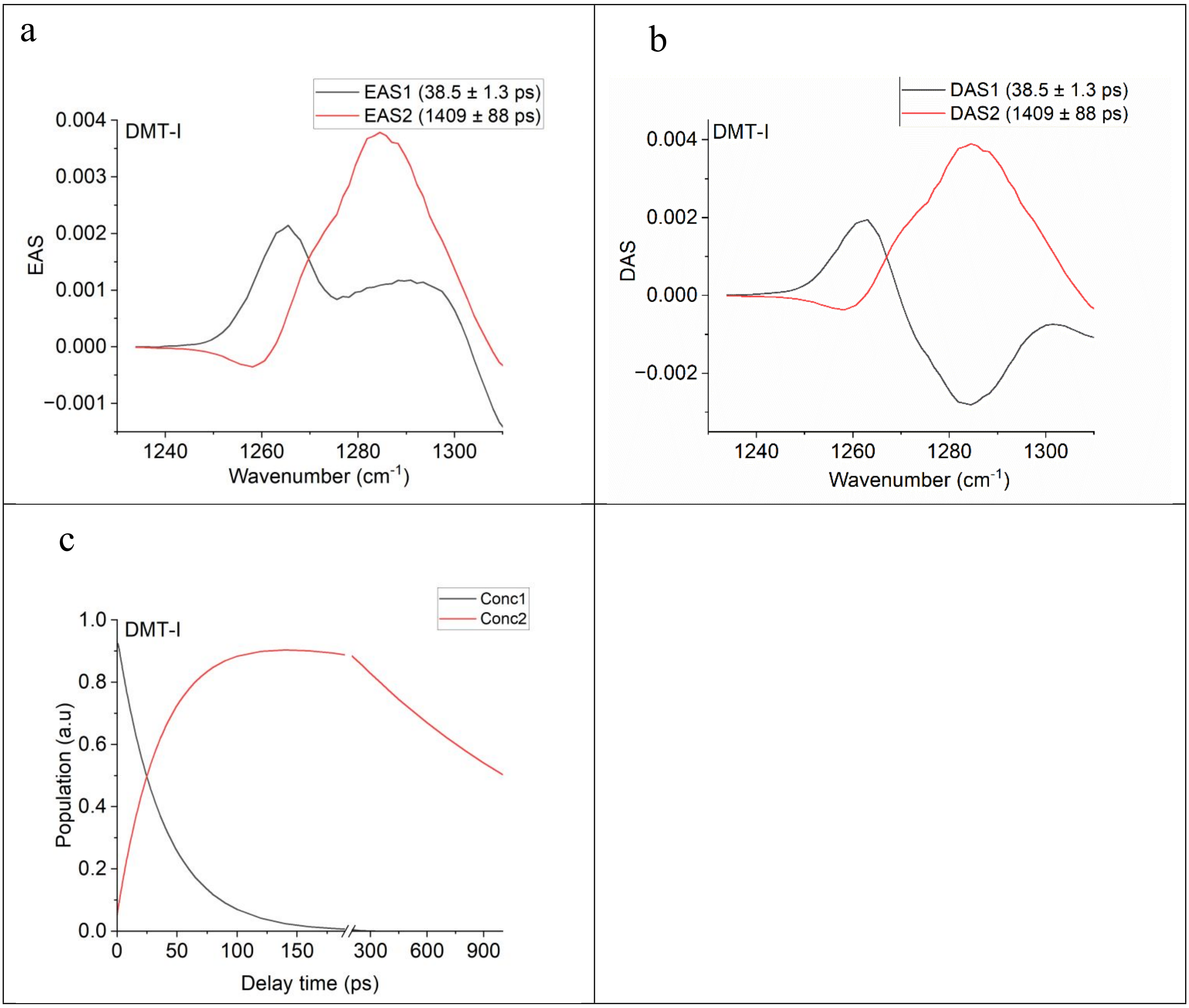

Fig. S3 Global analysis of transient absorption measurements of DMT-I in the experiments employing UV-Vis pump and IR probe, a) Decay-associated spectra, b) Evolution-associated spectra, c) Concentration profiles of transient species

b) DMT-M

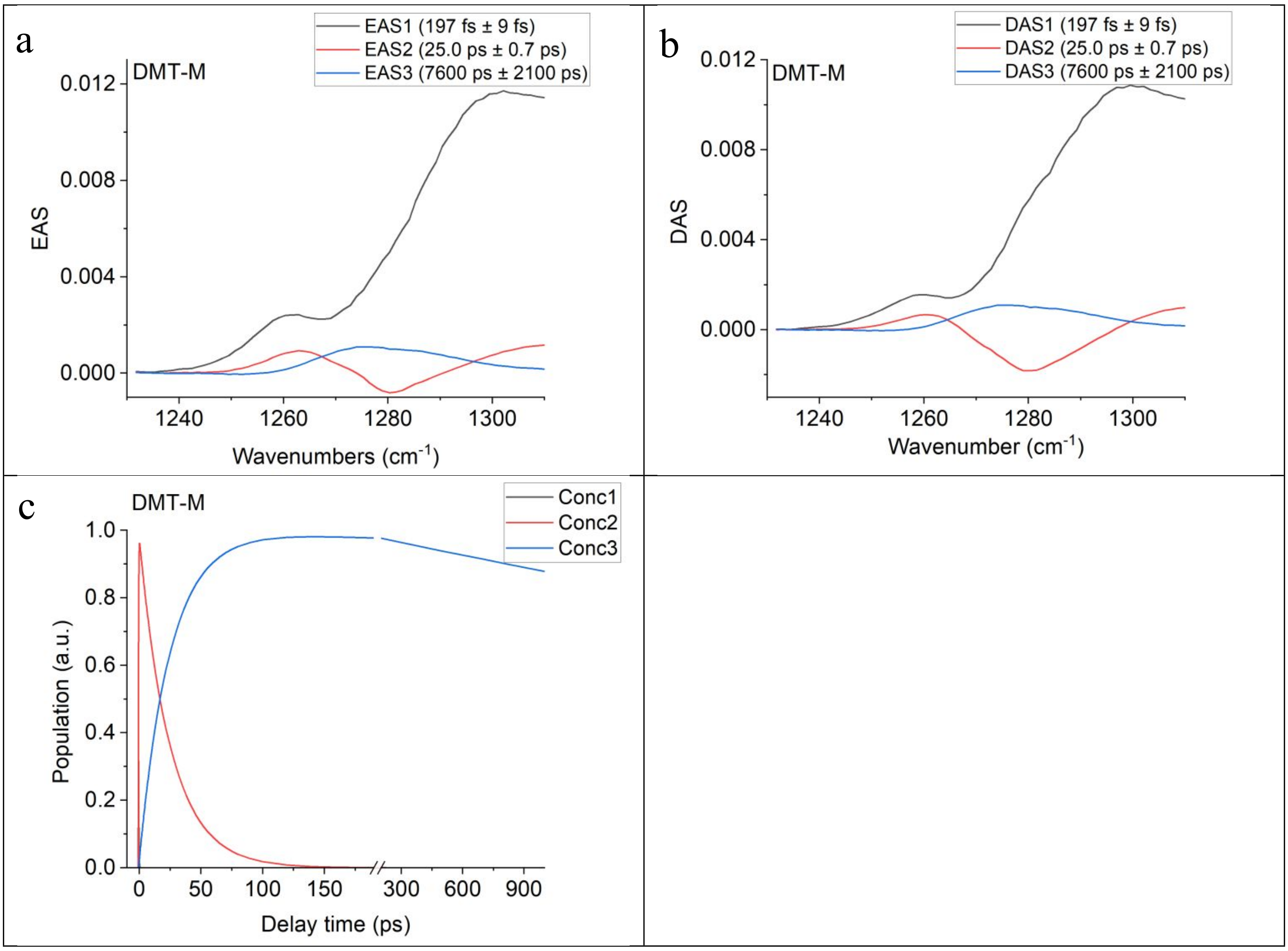

Fig. S4 Global analysis of transient absorption measurements of DMT-M in the experiments employing UV-Vis pump and IR probe, a) Decay-associated spectra, b) Evolution-associated spectra, c) Concentration profiles of transient species

c) DMT-N

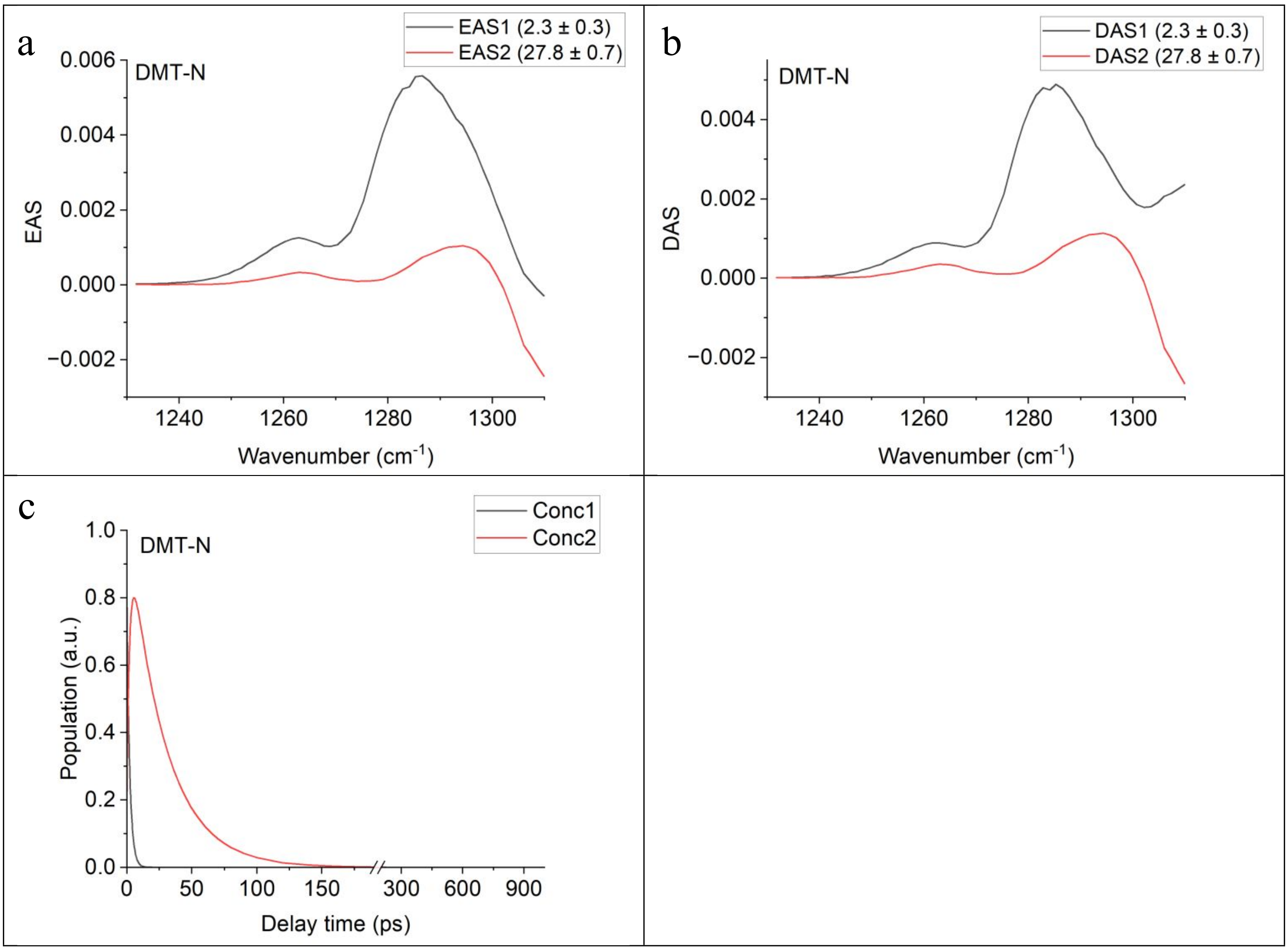

Fig. S5 Global analysis of transient absorption measurements of DMT-N in the experiments employing UV-Vis pump and IR probe, a) Decay-associated spectra, b) Evolution-associated spectra, c) Concentration profiles of transient species

3. Uncertainties of quantities determined through non-adiabatic dynamics simulation

	Final R_{cc} (Å)	Experimental st. dev. of R_{cc} (Å)	Degrees of freedom	Coverage factor for *p*=0.05	Expanded uncertainty (Å)
DMT-I	1.9420	0.0043	29	2.04	0.0087
DMT-M	2.0060	0.0042	50	2.01	0.0085
	τ_{CI} (fs)	Fitting error	Degrees of freedom	Coverage factor for *p*=0.05	Expanded uncertainty (fs)
DMT-I	120	Too few points to reliably fit a curve, maximum time taken as a boundary			
DMT-M	128.950	0.802	50	2.01	1.604

4. Geometrical rearrangements for vibrations in the 1250–1300 cm^{-1} range in DMT-I, DMT-M, and DMT-N, as determined through computations at the DFT/PBE0-D3 level.

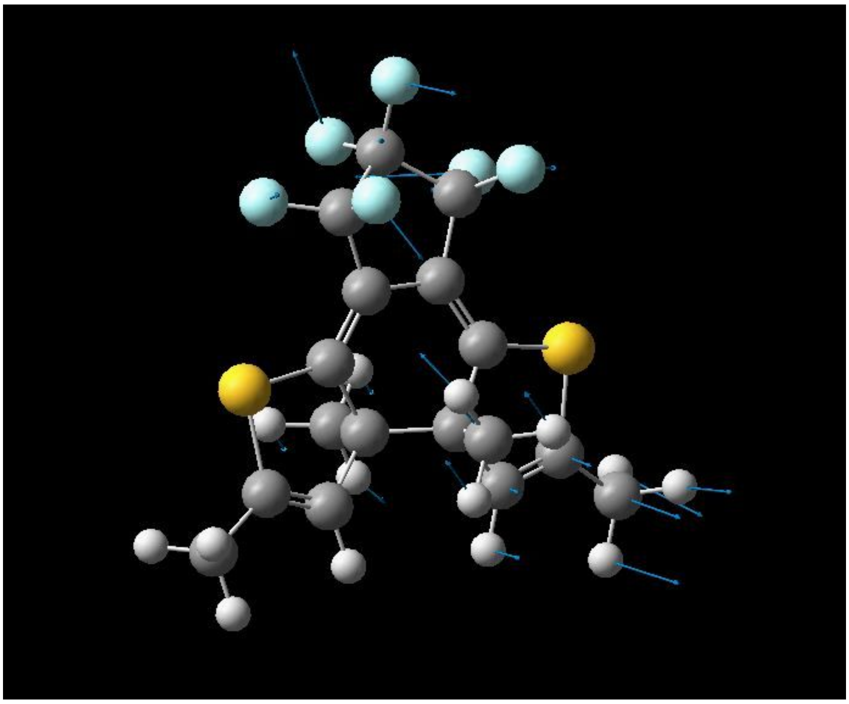

Fig. S6 DMT-I (theoretical frequency 1291 cm^{-1}, experimental frequency 1274 cm^{-1})

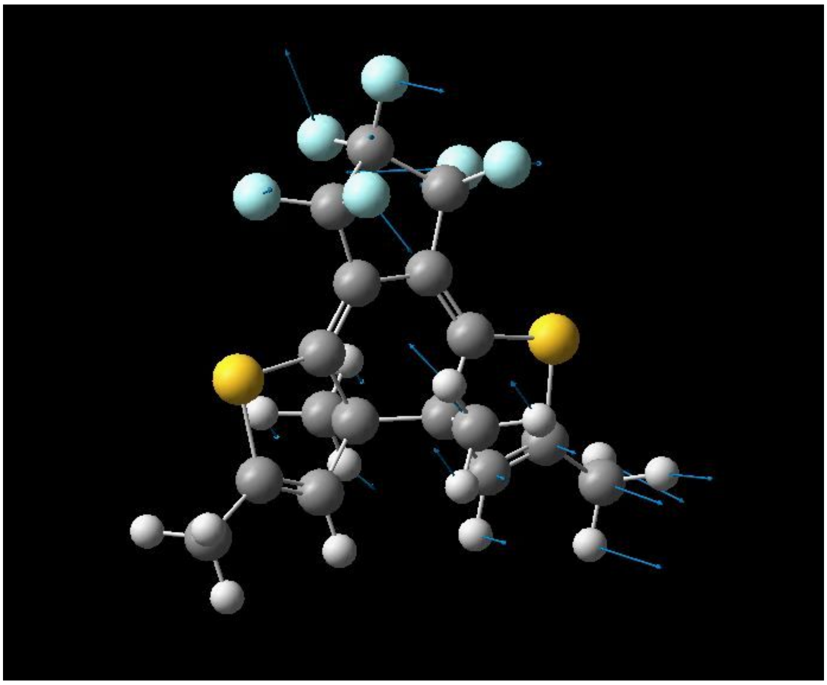

Fig. S7 DMT-M (theoretical frequency 1273 cm^{-1}, experimental frequency 1274 cm^{-1})

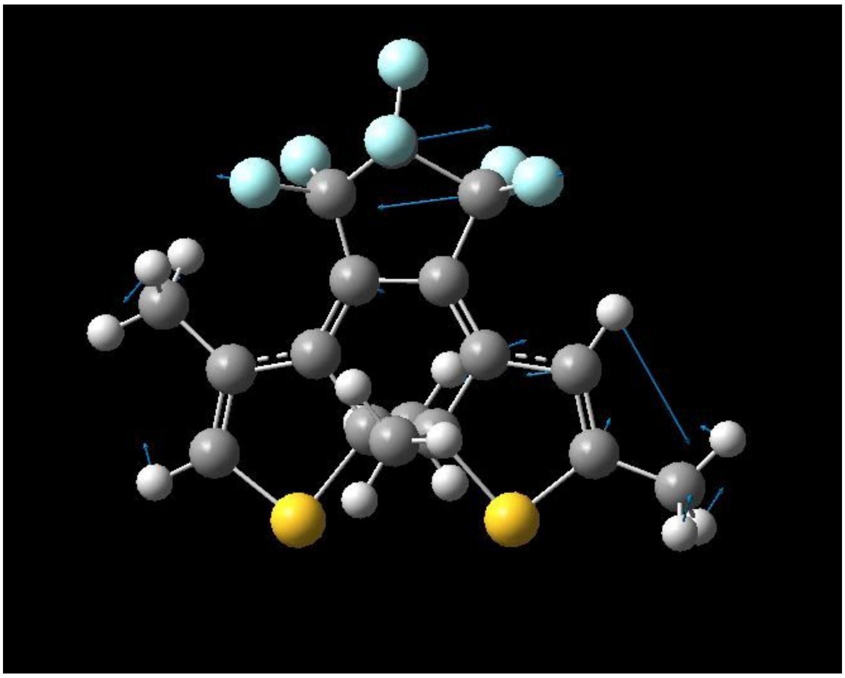

Fig. S8 DMT-N (theoretical frequency 1273 cm^{-1}, experimental frequency 1265 cm^{-1})

References

1) Snellenburg, J. J.; Laptenok, S.; Seger, R.; Mullen, K. M.; van Stokkum, I. H. M. Glotaran: A Java-Based Graphical User Interface for the R Package TIMP. *J. Stat. Soft.* **2012**, *49* (3), 1–22. https://doi.org/10.18637/jss.v049.i03.
